# Network-based statistics reveals an enhanced subnetwork in prefrontal cortex in mild cognitive impairment: a functional near-infrared spectroscopy study

**DOI:** 10.3389/fnagi.2024.1416816

**Published:** 2024-11-01

**Authors:** Peirong Wu, Zeping Lv, Yinuo Bi, Yijiang Li, Hong Chen, Jianfan Jiang, Suyan Pang, Xin Zhao, Wenyu Jiang

**Affiliations:** ^1^Department of Neurological Rehabilitation, Jiangbin Hospital of Guangxi Zhuang Autonomous Region, Nanning, China; ^2^Beijing Key Laboratory of Rehabilitation Technical Aids for Old-Age Disability, National Research Center for Rehabilitation Technical Aids, Beijing, China; ^3^Cognitive Rehabilitation Center, Jiangbin Hospital of Guangxi Zhuang Autonomous Region, Nanning, China; ^4^Faculty of Science and Engineering, University of Nottingham Ningbo, Ningbo, China

**Keywords:** mild cognitive impairment, functional near-infrared spectroscopy, functional connectivity, network-based statistics, prefrontal cortex

## Abstract

**Background:**

Mild cognitive impairment (MCI) is generally considered to have a high risk of progression to Alzheimer’s disease. Our study aimed to investigate the abnormal functional connectivity (FC) in prefrontal cortex (PFC) in patients with MCI and explore the relationship between the observed changes and cognitive function.

**Methods:**

Sixty-seven patients with MCI and 71 healthy individuals were recruited for this study. All participants underwent the Montreal Cognitive Assessment (MoCA) and functional near-infrared spectroscopy (fNIRS) examinations.

**Results:**

Compared with healthy controls (HC), the patients with MCI exhibited significantly lower MoCA scores (*p* < 0.001). Through FC analysis, an enhanced subnetwork was observed in the right prefrontal cortex of the MCI group, covering four pairs of channel connections: CH12-CH15, CH12-CH16, CH13-CH15, and CH13-CH16. Moreover, the FC values of these four channel pairs and the education duration were significantly correlated with MoCA scores. Subsequently, a multiple linear regression model was performed to observe the independent factors of cognition decline, serving the education duration and the average FC values of subnetwork as independent variables and the MoCA scores as the dependent variable. The regression model showed a total of 25.7% explanation power (adjusted R^2^ = 0.257, *F* = 24.723, *p* < 0.001).

**Conclusion:**

Our study suggested that the enhanced subnetwork within the right PFC may be involved in the pathophysiology of MCI and serve as a potential target for the treatment of MCI.

## 1 Introduction

According to surveillance data published in *Lancet Public Health* in 2020 (Jia et al., 2020), the estimated prevalence of mild cognitive impairment (MCI) in China is 15.54%, affecting over 38 million individuals. MCI serves as an intermediate stage between normal aging and dementia, characterized by a more pronounced cognitive impairment beyond age-related decline, but not meeting the diagnostic criteria for dementia (Petersen et al., 1997). Importantly, MCI represents a noteworthy period that researchers and clinicians perceive as an opportunity for early diagnosis and proactive intervention to slow down the progression of dementia. This perspective highlights the significance of timely intervention during the MCI stage to prevent further cognitive decline in patients and provide opportunities for disease management and targeted therapeutic strategies.

The prefrontal cortex (PFC) is known to be crucially involved in multiple cognition function, including decision-making (Hiser and Koenigs, 2018), cognitive control (Miller, 2000), executive function (Radel et al., 2017), attention and working memory (Bahmani et al., 2019). The PFC dysfunction may lead to cognitive impairment. Patients with MCI have been found to display reduced increases in prefrontal oxygenated hemoglobin during delayed verbal recall task (Uemura et al., 2016) and the 0-back and 1-back tasks (Niu et al., 2013) compared to the healthy controls (HCs). However, Takahashi et al. (2022) utilized fNIRS to examine the hemodynamic response of the PFC during category fluency and finger tapping tasks, but found no discrepancy in oxygenated hemoglobin signals between the MCI patients and HCs. In the study of brain connectivity, Bu et al. (2019) observed a downward trend in brain connectivity in patients with MCI, specifically characterized by connections from the right prefrontal cortex to the left prefrontal cortex, as well as from the prefrontal cortex to the occipital cortex, although it did not reach statistical significance. However, the impact of PFC function on the development of MCI remains unclear, and there is also controversy regarding the causal relationship between PFC function and MCI.

Brain functional connectivity (FC) refers to the coordinated and synchronized neural activity signals among different brain regions, unveiling the intricate interplay between these regions. Resting-state functional connectivity (RSFC), specifically, refers to the correlations observed in low-frequency oscillations between brain regions during a resting-state condition (van den Heuvel et al., 2009). RSFC has become a valuable tool for exploring complex FC patterns in various neurological and psychiatric disorders, yielding valuable insights into their underlying mechanisms. Researchers often employ traditional multiple comparison methods, such as the false discovery rate (FDR) correction and Bonferroni correction, to detect intergroup differences. These methods involve evaluating the strength of FC for each connection by computing independent test statistics and their corresponding *p*-values. However, this approach necessitates a large number of multiple comparisons, leading to a diminished statistical power and an elevated risk of false negatives. To address these limitations, a multivariate testing method called network-based statistics (NBS) has been employed. NBS focuses on network components and identifies interconnected structures or components that exhibit interconnections surpassing a specified threshold (Zalesky et al., 2010). The significance of each identified component is evaluated by comparing its size to the null distribution of the maximum component size, thereby assigning a *p*-value to each component. NBS has proven effective in identifying dysfunctional brain connections and has been applied in the investigation of various neurological and psychiatric disorders (Zajac et al., 2017; Lopes et al., 2017).

Over the past few years, functional near-infrared spectroscopy (fNIRS) has gained significant recognition as a non-invasive neuroimaging technique in the field of neurobiology. Compared to fMRI, fNIRS offers several advantages, including cost-effectiveness, portability, lower sensitivity to motion artifacts, and higher temporal sampling rate. By utilizing optically sensitive probes placed on the scalp, fNIRS allows for the emission and detection of near-infrared light, enabling the quantification of oxyhemoglobin (HbO) and deoxyhemoglobin (HbR) concentrations within the cortical regions of the brain (Yeung and Chan, 2020). Therefore, fNIRS offers a practical and effective tool for investigating brain activity, facilitating insights into the functions and mechanisms of the brain.

In a study based on fNIRS and connectivity neurofeedback, the feedback signal was derived from the strength of frontal–parietal functional connections, with working memory as the targeted cognitive function (Xia et al., 2021). The study consisted of three sessions of approximately 15 min each, wherein healthy participants underwent neurofeedback training. The results revealed a significant increase in functional connectivity among healthy participants after the training, accompanied by notable improvements in cognitive functions such as memory and attention. However, there is a lack of research on neurofeedback training methods focusing on patients with MCI, and the existing studies often restrict the feedback signal to the functional connectivity values of individual connections, neglecting investigations into network connectivity modulation related to cognitive impairments. This study aims to investigate the abnormal functional connectivity in patients with MCI, focusing on the closely related prefrontal lobe, to identify crucial target regions for network connectivity neurofeedback.

## 2 Materials and methods

### 2.1 Participants

A total of 164 participants were enrolled from local communities in Nanning City, China. After excluding participants who did not complete the cognitive assessments and those with data quality issues (channel signal coefficient of variation >15%), a final sample of 138 participants was included. The final sample comprised 67 patients with MCI and 71 age-, gender-, and education-matched healthy individuals. Cognitive assessments were conducted using the Chinese version of the Montreal Cognitive Assessment (MoCA), with a maximum score of 30. For participants with <12 years of education, 1 point was added to their MoCA total score.

All participants met the following inclusion criteria: (1) aged 55 years or above; (2) no clinical diagnosis of AD; (3) right-handedness. In addition to these criteria, MCI patients had to meet the following requirements: (1) presence of subjective memory complaints; (2) according to Chinese MoCA norms (Lu et al., 2011), MoCA scores of ≤13 for illiterate individuals, ≤19 for individuals with 1–6 years of education, and ≤ 24 for individuals with 7 or more years of education.

Exclusion criteria consisted of: (1) comorbid psychiatric or neurological disorders, as well as other systemic diseases; (2) severe impairments in vision or hearing, or conditions such as age-related cataracts that would hinder participant cooperation; (3) history of using antipsychotic medications or substance abuse.

This study was approved by the Ethics Committee of Jiangbin Hospital, Guangxi Zhuang Autonomous Region. All participants included in the study provided informed consent prior to enrollment.

### 2.2 Data acquisition

**Figure 1:**
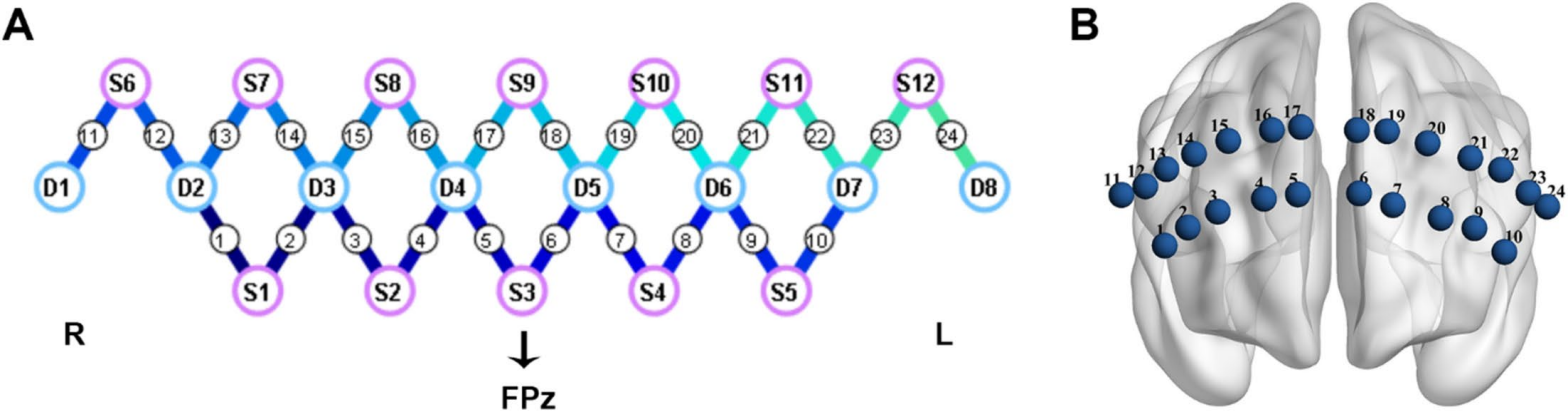
Cap configuration. **(A)** The purple circles represent light sources; The blue circles represent detectors; the connecting lines between sources and detectors represent measured channels. **(B)** The layout diagram of 24 channels on the prefrontal region. L, left; R, right.

This study utilized a portable fNIRS device (NirSmart, HuiChuang, China) to measure the hemodynamic responses in the PFC. The system operated at a sampling rate of 10 Hz, with wavelengths of 760 nm and 850 nm. The device was equipped with 12 light sources and 8 detectors, resulting in a total of 24 channels (refer to Figure 1). The precise mapping of brain regions corresponding to these 24 channels could be found in Supplementary Table 1. The placement of the probes followed the internationally recognized 10/20 electrode placement system. The central position of light source S3 was designated as the FPz channel, while the remaining lower probes were carefully aligned along the Fp1-Fp2 line, ensuring accurate and standardized placement.

After completing the collection of personal information and MoCA assessments, participants were given a 10-min rest period before initiating the acquisition of fNIRS data. During the study, fNIRS signals were collected from participants during an 8-min resting period. Throughout the data acquisition process, participants were instructed to maintain a comfortable sitting position, close their eyes, relax, and avoid any excessive head movements.

### 2.3 Data preprocessing and channel-based FC analysis

The preprocessing of fNIRS data was carried out using the Homer2 toolkit on the Matlab 17.0 platform (Huppert et al., 2009). Initially, the raw signals were converted into optical density using the “hmrIntensity2OD” function. Motion artifacts were then eliminated using the “hmrMotionArtifactByChannel” and “hmrMotionCorrectSpline” functions, with the parameters set as follows: STDEVthresh = 30.0, AMPThresh = 3.00, tMotion = 1 s, tMask = 1 s, and *p* = 0.99. Subsequently, a band-pass filter was applied within the frequency range of 0.01 Hz to 0.1 Hz using the “hmrBandpassFilt” function. Finally, the concentrations of HbO and HbR were derived from the optical density using the “hmrOD2Conc” function, with a differential pathlength factor set to 6.0. Since HbO is a more reliable indicator of changes in cerebral cortex blood flow (Hoshi, 2007) and exhibits a higher signal-to-noise ratio compared to HbR (Tong and Frederick, 2010), it was selected for further analysis.

The Pearson correlation coefficient was calculated to assess the FC values between pairs of measurement channels, yielding a 24 × 24 correlation matrix for each participant. Following this, Fisher’s r-to-z transformation was applied to convert the correlation coefficients into z-scores in order to improve the normality properties of the data.

### 2.4 Statistics analysis

The statistical analysis of clinical data and MoCA scores was conducted using SPSS 17.0 (SPSS Inc., Chicago, IL, USA). The normality of the quantitative data was initially assessed using the Kolmogorov–Smirnov test. Subsequently, intergroup comparisons were conducted based on the distribution of the data. Specifically, independent t-tests were employed for data with a normal distribution, while the Mann–Whitney *U-*test was utilized for data that did not follow a normal distribution. The gender variable was analyzed using the chi-squared test. Statistical significance was defined as *p* < 0.05.

For the fNIRS data, we utilized two different methods to perform statistical tests in order to identify differences in prefrontal FC pattern between the two groups. The first method employed independent samples *t*-tests with FDR correction, and a significance level of *p* < 0.05 was used to indicate a statistical difference. The second method involved employing the NBS approach, implemented using the NBS 1.2 toolbox in Matlab 17.0. NBS utilizes a cluster-based thresholding methodology to address the issue of multiple comparisons by treating clusters of connected components in the network as a single entity. For threshold selection, we referred to a *t*-value probability table and chose the *t*-value corresponding to *p* = 0.01 and degrees of freedom >120, which is 2.6. A *t*-test was performed on each connection, with a *p*-value threshold of 0.05 and 5,000 permutations. The magnitude of significant components was measured based on extent (Riccardi et al., 2023).

Moreover, the average FC values of channel pairs displaying significant variations were extracted. Pearson correlation analyses were conducted to investigate the associations between the FC values and the MoCA scores. Given the non-normal distribution of education duration data among the participants, Spearman’s method was utilized to perform a correlation analysis with the MOCA scores. Furthermore, multiple linear regression analysis was employed to explore the independent factors influencing the MoCA scores, with the significantly correlated variables serving as independent variables and the MoCA scores as the dependent variable.

## 3 Results

### 3.1 Demographic and clinical characteristics

No significant statistical difference was found in gender, age, and education level between the two groups (*p* > 0.05). The MCI group had significantly lower MoCA total scores compared to the HC group (*p* < 0.001). Please refer to Table 1 for detailed results.

**Table 1:** Comparison of clinical data and MoCA scores between the two groups.

Characteristics	MCI group	HC group	*p* value
Gender (M/F)	32/35	40/31	0.313^a^
Age (years)	69.40 ± 5.50	70.82 ± 4.71	0.107
Education (years)	9.13 ± 3.99	9.42 ± 4.16	0.679
MoCA total scores	18.76 ± 3.39	24.63 ± 2.49	< 0.001^*^

MCI, mild cognitive impairment; HC, heathy control; MoCA, Montreal Cognitive Assessment; ^a^*χ*^2^ test for sex (*n*). Independent *t*-test for the other data (means ± SD). *p < 0.05 indicates statistical significance.

### 3.2 FC analysis

**Figure 2:**
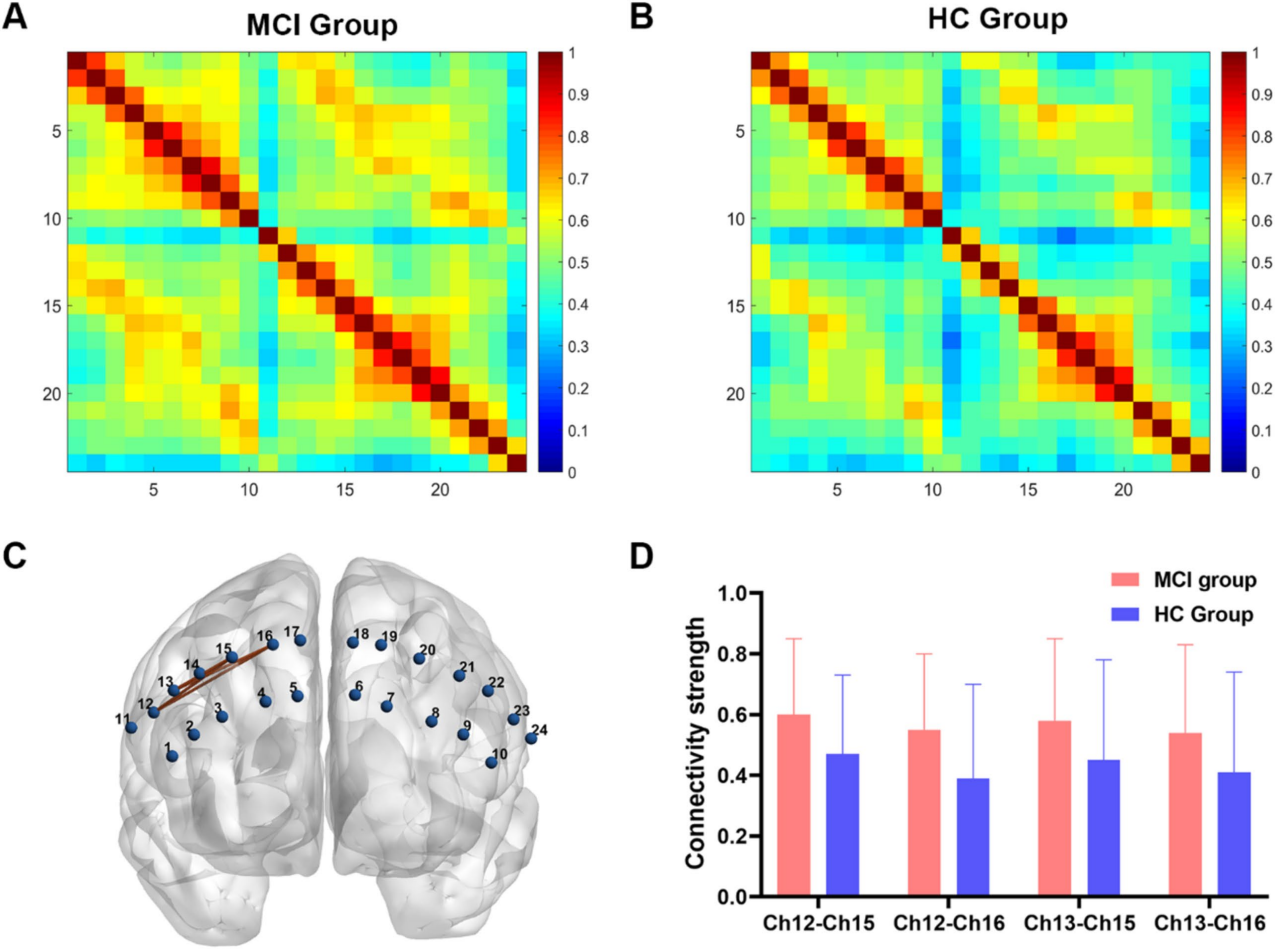
Functional connectivity analysis between two groups. **(A,B)** Connectivity matrices of 24 channels in the MCI group and HC group. **(C)** An enhanced subnetwork in the prefrontal cortex was identified using the network-based statistic method. **(D)** Bar chart showed the differences in the within-subnetwork connectivity of two groups (*p* < 0.05). MCI, mild cognitive impairment; HC, healthy control.

In the FC analysis, two HbO correlation matrix maps were generated for the two groups (Figures 2A,B). According to the traditional multiple comparison results, the MCI group displayed increased connectivity strength in two channel pairs compared to the HC group: Ch12-Ch15 and Ch12-Ch16 (pars opercularis of right Broca’s area - right dorsolateral prefrontal cortex) (FDR corrected, *p* < 0.05).

However, the NBS analysis revealed that in addition to the aforementioned two channel pairs, the MCI group also showed significantly increased connectivity strength in Ch13-Ch15 and Ch13-Ch16 (pars triangularis of right Broca’s area - right dorsolateral prefrontal cortex) (Figures 2C,D). In the MCI group, no channel pairs were identified to exhibit lower FC values compared to the HC group. The details of NBS result were shown in Table 2.

**Table 2:** Channel pairs with significant differences in connectivity strength between groups based on NBS (mean ± SD).

Channel pairs	Brain regions	Connectivity strength		*t*	*p*
		MCI group	HC group		
Ch12-Ch15	pars opercularis of Broca	0.60 ± 0.25	0.47 ± 0.26	3.02	0.033
Ch12-Ch16	mirror area – rDLPFC	0.55 ± 0.25	0.39 ± 0.31	3.45	
Ch13-Ch15	pars triangularis of Broca	0.58 ± 0.27	0.45 ± 0.33	3.23	
Ch13-Ch16	mirror area – rDLPFC	0.54 ± 0.29	0.41 ± 0.33	3.68	

NBS, network based statistic; SD, standard deviation; MCI, mild cognitive impairment; HC, healthy control; rDLPFC, right dorsolateral prefrontal cortex.

### 3.3 Multiple linear regression analysis

**Figure 3:**
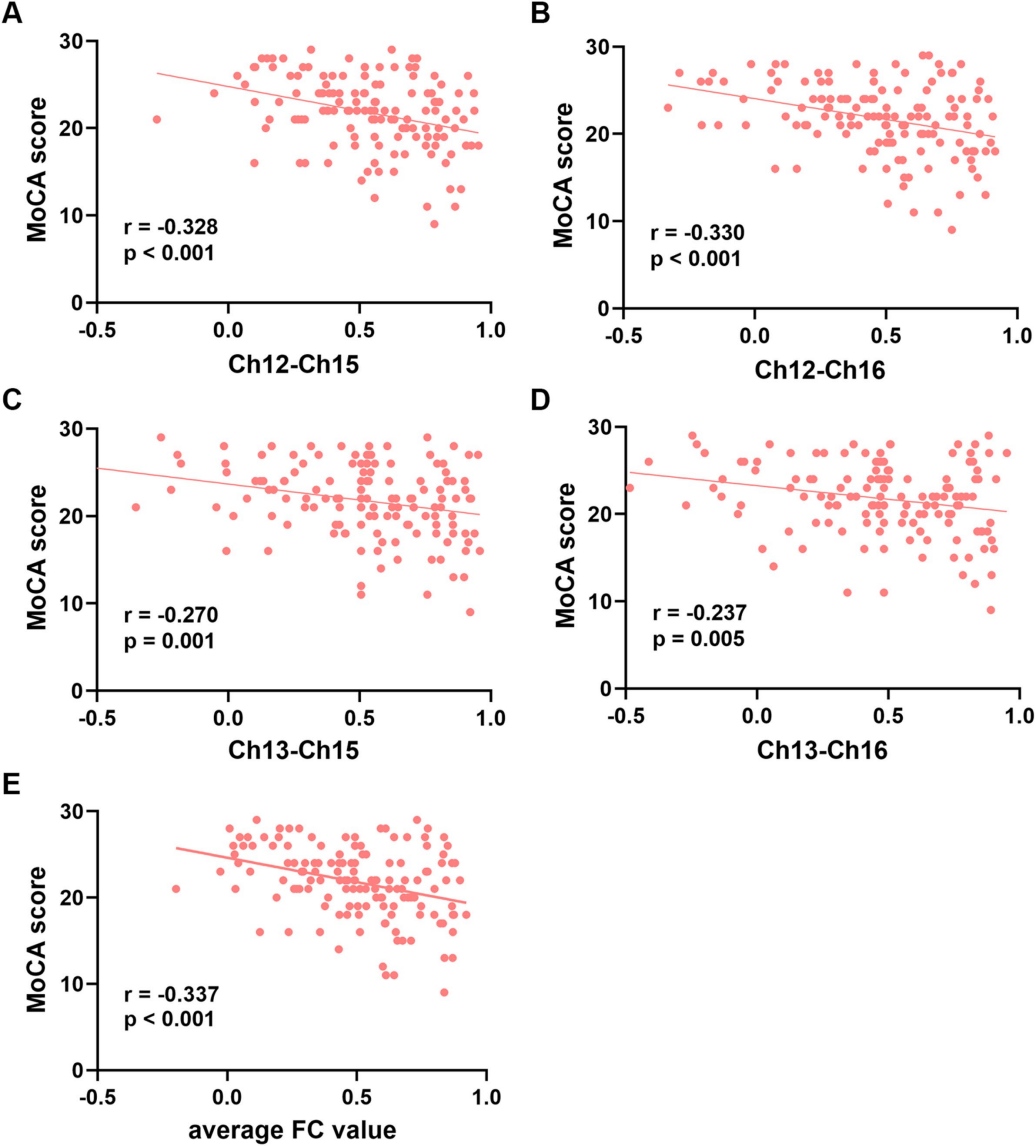
Correlation analysis between the functional connectivity values of the four channel pairs and MoCA scores. **(A-D)** The FC values of the channel pairs Ch12-Ch15, Ch12-Ch16 Ch13-Ch15 and Ch13-Ch16 were significantly correlated with MoCA scores (all *p* < 0.05). **(E)** The average FC values of the four channel pairs were correlated with MoCA scores (*p* < 0.001). Ch, channel; MoCA, Montreal Cognitive Assessment; FC, functional connectivity.

The FC values of the channel pairs Ch12-Ch15 (*r* = −0.328, *p* < 0.001), Ch12-Ch16 (*r* = −0.330, *p* < 0.001), Ch13-Ch15 (*r* = −0.270, *p* = 0.001) and Ch13-Ch16 (*r* = −0.237, *p* = 0.005), along with education duration (*r* = 0.365, *p* < 0.001), exhibited significant correlation with MoCA scores (Figures 3A–D). No significant correlation was found between age and MoCA scores (*p* > 0.05). Therefore, the education duration and the FC values of these four channel pairs were treated as independent variables, while the MoCA scores considered the dependent variable for the multiple linear regression model. As shown in Table 3, this model (Model 1) yielded significant results, explaining 26.6% of the variance (adjusted R^2^ = 0.266, *F* = 10.944, *p* < 0.001). Specifically, the education duration demonstrated a significant positive effect on MoCA scores (*b* = 0.409, *t* = 3.011, *p* < 0.001). In addition, the FC values of four channel pairs had no significant effect on the MoCA score (*p* > 0.05).

**Table 3:** Multiple linear regression results with education and FC values of four channel pairs as independent variables, and MoCA scores as dependent variable.

Education and FC values → MoCA	Regression coefficients				Model summary		
	Unstd B	Std B	*t*	*p*	Adjusted R^2^	F	*p*
Model 1							
Education	0.409	0.399	3.011	<0.001^*^	0.266	10.944	<0.001^*^
FC values							
Ch12-Ch15	−2.827	−0.167	−1.277	0.204			
Ch12-Ch16	−2.322	−0.160	−1.204	0.231			
Ch13-Ch15	−3.367	−0.249	−1.623	0.107			
Ch13-Ch16	2.307	0.176	1.099	0.274			
Model 2							
Education	0.403	0.393	5.338	<0.001^*^	0.257	24.723	<0.001^*^
Average FC values	−5.521	−0.328	−4.460	<0.001^*^			

FC, functional connectivity; MoCA, Montreal Cognitive Assessment; Unstd, Un standardized; std, standardized; B, Beta; R^2^ = R-squared (coefficient of determination); ^*^p < 0.05 indicates statistical significance.

Next, we attempted to determine the average FC values of the four pairs of channels and conducted a Pearson correlation analysis with the MoCA scores. We found that the average FC values were also negatively correlated with MoCA scores (*r* = −0.337, *p* < 0.001, Figure 3E). The second multiple linear regression model was conducted, with education and the average FC values of the four channel pairs as independent variables, and MoCA scores as the dependent variable. As shown in Table 3, this model (Model 2) yielded significant results, explaining 25.7% of the variance (adjusted R^2^ = 0.257, *F* = 24.723, *p* < 0.001). Specifically, education duration and the average FC values demonstrated a significant positive effect on MoCA scores (*b* = 0.409, *t* = 3.011 and *b* = −5.521, *t* = −4.460, respectively, *p* < 0.001). The standardized regression coefficients for these factors were 0.393 and −0.328, respectively. These results suggested that the influence of the subnetwork in the right PFC on MoCA scores is comparable to that of education.

## 4 Discussion

The PFC is a crucial region of the human brain that plays a significant role in many cognitive functions, emotional regulation, and decision-making processes. Investigating the aberrant changes in FC patterns in the prefrontal cortex of patients with MCI and understanding their relationship with cognitive impairment can contribute to the development of more identification of potential treatment targets. With these objectives in mind, our study aimed to identify abnormal prefrontal connectivity patterns in patients with MCI and compare the statistical power of traditional multiple comparison methods with the NBS method. Based on the results of FC analysis, two multiple linear regression model were conducted to investigate the associates of cognitive impairment.

In this study, an abnormal subnetwork was identified in the right PFC of the MCI group using the NBS method. This subnetwork consisted of four pairs of channel connections (Ch12-Ch15, Ch12-Ch16, Ch13-Ch15, and Ch13-Ch16). Traditional multiple comparison methods such as false discovery rate (FDR) correction and Bonferroni correction are commonly employed to detect group differences by assessing the strength of individual connections through test statistics and *p*-values. However, these methods necessitate correction for multiple comparisons, which can reduce statistical power and increase the risk of false negatives. In contrast, NBS adopts a multivariate approach that focuses on analyzing interconnected structures or components within networks rather than individual connections. This approach enhances statistical power by evaluating entire network components collectively. The application of NBS in this study thus provided a robust methodological framework compared to traditional multiple comparison approaches. Previous research has similarly highlighted NBS’s efficacy in identifying altered subnetworks associated with neurodegenerative disorders, underscoring its utility in network analyses within neuroscientific research.

Several studies have shown that in patients with MCI, reduced hippocampal volume is associated with increased connectivity in the PFC, suggesting that this heightened connectivity may serve as a compensatory mechanism in response to cognitive challenges (Agosta et al., 2010; Kircher et al., 2007; Clément et al., 2013). Similar to our findings, Nguyen et al. (2019) also observed increased connectivity in the right PFC in patients with MCI, surpassing that of the normal population. Additionally, healthy aging was characterized by an increase connectivity in frontal lobe and a decrease in connectivity within the posterior default mode network (DMN). These changes were more prominent in AD, indicating an accelerated aging pattern (Jones et al., 2011). The increased connectivity in the PFC in AD/MCI was consistently observed and was often attributed to compensatory mechanisms for disrupted connections in other brain regions (Krajcovicova et al., 2014). To compensate for decreased processing efficiency, individuals with MCI may need to enhance the functioning of specific regions to achieve a level of performance comparable to that of healthy individuals. However, as the disease progresses, these compensatory mechanisms may become less effective, leading to a decline in interregional connectivity (Supekar et al., 2008; Esposito et al., 2013). These changes in connectivity patterns over time highlighted the dynamic nature of brain networks during the progression of neurodegenerative diseases. Consequently, the heightened connectivity within prefrontal subnetwork in this study may also reflect cognitive compensation.

The subnetwork establishes connections between the right dorsolateral prefrontal cortex (DLPFC) and right Broca mirror area. The right DLPFC plays a crucial role executive control processes, especially when dealing with interference information (Anderson et al., 2004). Excessive activity in this region has been identified as one of the functional brain abnormalities associated with memory impairment in patients with MCI/AD (Wang et al., 2006). When individuals actively use cognitive resources to suppress distracting information, the right DLPFC is activated (Diekelmann et al., 2011). Conversely, negative activation of the right DLPFC had been correlated with improved memory performance and reduced competition from memory interference (Kuhl et al., 2007). Turriziani et al. (2012) found that excitatory transcranial magnetic stimulation applied to the right DLPFC impeded subsequent declarative memory retrieval, while inhibitory transcranial magnetic stimulation facilitated retrieval. Similarly, studies involving transcranial direct current stimulation conducted by Au et al. (2021) supported the role of the right DLPFC in memory interference during memory reactivation or consolidation phases. The right Broca mirror area refers to the corresponding area in the right hemisphere that is structurally and functionally analogous to the left Broca’s area. While the left Broca’s area is traditionally associated with language production and speech fluency, the right homologous area has been implicated in various functions, including prosody, emotional expression and the application of language (Mitchell et al., 2003; Meyer et al., 2002), concentration (Magan and Yadav, 2021) and cognitive inhibition (Aron et al., 2004). As the left hemisphere is typically dominant for language functions, increased connectivity between the right DLPFC and the Broca mirror area may reflect an adaptive response to compensate for potential deficits in the left hemisphere. Similar to the earlier interpretation, the observed connectivity changes may signify network reorganization or adaptive changes in response to cognitive decline in MCI. The right hemisphere may be engaging in compensatory processes or assuming a more prominent role in language processing and executive function.

To further explore the potential role of the right hemisphere in cognitive compensation, future research should employ advanced brain imaging techniques and behavioral assessment methods to analyze the activity patterns of the right hemisphere during specific cognitive tasks. Additionally, it is essential to investigate how the right hemisphere achieves effective compensation in the context of brain injury or other cognitive disorders. This will provide important insights for our understanding of brain plasticity and its role in recovery and adaptation.

Moreover, our study employed a multiple linear regression model, which demonstrated a significant association between the duration of education and the average FC value of a specific subnetwork, with MoCA scores. This finding suggests that individuals with a higher level of education or lower subnetwork connections tend to exhibit higher MoCA scores. This could indicate that educational attainment may foster cognitive resilience, enabling individuals to achieve better cognitive performance. Additionally, lower subnetwork connections may indicate a more efficient information processing approach in cognitive tasks. However, it is important to note that the regression model only explains a portion of the variance in MoCA scores, which may be due to the inclusion of a limited number of variables. Further research is needed to explore additional factors that may contribute to the variance in MoCA scores, such as genetic variations, lifestyle choices, and psychological status. It would also be beneficial to investigate the interaction effects of these variables with education on cognitive performance.

The present study utilized fNIRS technology to investigate abnormal functional connectivity in the PFC in individuals with MCI, aiming to uncover the underlying pathological mechanisms associated with the occurrence of MCI, and to facilitate the development of more accurate diagnostic tools and effective treatment methods. In summary, the observed increased connectivity between the right DLPFC and the right Broca mirror area in MCI patients highlights the potential involvement of the right hemisphere in compensatory mechanisms or adaptive processes related to language processing and executive function. This finding may lay the foundation for exploring the cognitive strategies of MCI patients from a neural network perspective, contributing to a better understanding of how the brain adapts to and compensates for cognitive loss.

### 4.1 Limitations

Several limitations need to be acknowledged in this study. Firstly, the inclusion of participants from a specific region may introduce bias in sample selection, potentially restricting the generalizability of the findings to a wider population of patients with MCI. Secondly, the cross-sectional design of this study precludes the acquisition of long-term follow-up data on the progression of MCI. Future studies could consider employing longitudinal study designs to gain a better understanding of the pathological mechanisms and developmental trajectory of MCI. Thirdly, the use of fNIRS as a functional brain imaging tool may have inherent limitations, such as its relatively low spatial resolution and the inability to precisely localize specific brain regions. Furthermore, the limited penetration depth of light in fNIRS may not encompass the entire frontal cortex. To address these limitations, future studies could consider employing functional magnetic resonance imaging (fMRI), which offers superior spatial resolution, or electroencephalography (EEG), which provides enhanced temporal resolution. Utilizing these methodologies in conjunction may yield more comprehensive and accurate insights into brain function. Fourthly, considering that MCI involves widespread changes across the entire brain, a predominant focus on the PFC may overlook important information from other brain regions, thus limiting a holistic understanding of the mechanisms underlying MCI. Future research should broaden its scope to include as many brain regions as possible, allowing for a more comprehensive understanding of the pathophysiology of MCI.

## 5 Conclusion

Our study indicates that individuals with MCI exhibit enhanced connectivity within the PFC, which may be related to MCI pathophysiology or cognitive compensation. Furthermore, these findings suggest that alterations in PFC connectivity may serve as potential biomarkers for early detection and monitoring of MCI progression.

## Data Availability

The raw data supporting the conclusions of this article will be made available by the authors, without undue reservation.
