# Supplementary material for: Network-based statistics reveals an enhanced subnetwork in prefrontal cortex in mild cognitive impairment: a functional near-infrared spectroscopy study
Source: Front Aging Neurosci. 2024 Nov 1;16:1416816. doi: 10.3389/fnagi.2024.1416816 (PMC11565517; doi:10.3389/fnagi.2024.1416816)
Supplement: Supplementary file 1 [file Table_1.docx]

**Supplementary Table 1** The corresponding brain regions of 24 NIRS channels.

| Label of Channel | Brodmann Area | Percentage |
| --- | --- | --- |
| CH1 (S1-D2) | 45 - pars triangularis Broca's area | 0.7643 |
|  | 46 - Dorsolateral prefrontal cortex | 0.2357 |
|  |  |  |
| CH2 (S1-D3) | 45 - pars triangularis Broca's area | 0.3402 |
|  | 46 - Dorsolateral prefrontal cortex | 0.6598 |
|  |  |  |
| CH3 (S2-D3) | 10 - Frontopolar area | 0.0261 |
|  | 46 - Dorsolateral prefrontal cortex | 0.9739 |
|  |  |  |
| CH4 (S2-D4) | 9 - Dorsolateral prefrontal cortex | 0.392 |
|  | 10 - Frontopolar area | 0.404 |
|  | 46 - Dorsolateral prefrontal cortex | 0.204 |
|  |  |  |
| CH5 (S3-D4) | 9 - Dorsolateral prefrontal cortex | 0.4307 |
|  | 10 - Frontopolar area | 0.5693 |
|  |  |  |
| CH6 (S3-D5) | 9 - Dorsolateral prefrontal cortex | 0.4124 |
|  | 10 - Frontopolar area | 0.5876 |
|  |  |  |
| CH7 (S4-D5) | 9 - Dorsolateral prefrontal cortex | 0.2724 |
|  | 10 - Frontopolar area | 0.5331 |
|  | 46 - Dorsolateral prefrontal cortex | 0.1946 |
|  |  |  |
| CH8 (S4-D6) | 10 - Frontopolar area | 0.0531 |
|  | 46 - Dorsolateral prefrontal cortex | 0.9469 |
|  |  |  |
| CH9 (S5-D6) | 45 - pars triangularis Broca's area | 0.2988 |
|  | 46 - Dorsolateral prefrontal cortex | 0.7012 |
|  |  |  |
| CH10 (S5-D7) | 45 - pars triangularis Broca's area | 0.8811 |
|  | 46 - Dorsolateral prefrontal cortex | 0.1189 |
|  |  |  |
| CH11 (S6-D1) | 6 - Pre-Motor and Supplementary Motor Cortex | 0.6364 |
|  | 43 - Subcentral area | 0.2825 |
|  | 44 - pars opercularis_ part of Broca's area | 0.0812 |
|  |  |  |
| CH12 (S6-D2) | 6 - Pre-Motor and Supplementary Motor Cortex | 0.0526 |
|  | 44 - pars opercularis_ part of Broca's area | 0.6982 |
|  | 45 - pars triangularis Broca's area | 0.2491 |
|  |  |  |
| CH13 (S7-D2) | 9 - Dorsolateral prefrontal cortex | 0.034 |
|  | 44 - pars opercularis_ part of Broca's area | 0.3962 |
|  | 45 - pars triangularis Broca's area | 0.5434 |
|  | 46 - Dorsolateral prefrontal cortex | 0.0264 |
|  |  |  |
| CH14 (S7-D3) | 9 - Dorsolateral prefrontal cortex | 0.5392 |
|  | 44 - pars opercularis_ part of Broca's area | 0.0691 |
|  | 45 - pars triangularis Broca's area | 0.1843 |
|  | 46 - Dorsolateral prefrontal cortex | 0.2074 |
|  |  |  |
| CH15 (S8-D3) | 8 - Includes Frontal eye fields | 0.0089 |
|  | 9 - Dorsolateral prefrontal cortex | 0.9911 |
|  |  |  |
| CH16 (S8-D4) | 8 - Includes Frontal eye fields | 0.1 |
|  | 9 - Dorsolateral prefrontal cortex | 0.9 |
|  |  |  |
| CH17 (S9-D4) | 8 - Includes Frontal eye fields | 0.1967 |
|  | 9 - Dorsolateral prefrontal cortex | 0.8033 |
|  |  |  |
| CH18 (S9-D5) | 8 - Includes Frontal eye fields | 0.1303 |
|  | 9 - Dorsolateral prefrontal cortex | 0.8697 |
|  |  |  |
| CH19 (S10-D5) | 8 - Includes Frontal eye fields | 0.0667 |
|  | 9 - Dorsolateral prefrontal cortex | 0.9333 |
|  |  |  |
| CH20 (S10-D6) | 9 - Dorsolateral prefrontal cortex | 0.9823 |
|  | 46 - Dorsolateral prefrontal cortex | 0.0177 |
|  |  |  |
| CH21 (S11-D6) | 9 - Dorsolateral prefrontal cortex | 0.4306 |
|  | 44 - pars opercularis_ part of Broca's area | 0.0463 |
|  | 45 - pars triangularis Broca's area | 0.25 |
|  | 46 - Dorsolateral prefrontal cortex | 0.2731 |
|  |  |  |
| CH22 (S11-D7) | 44 - pars opercularis_ part of Broca's area | 0.419 |
|  | 45 - pars triangularis Broca's area | 0.581 |
|  |  |  |
| CH23 (S12-D7) | 6 - Pre-Motor and Supplementary Motor Cortex | 0.1601 |
|  | 44 - pars opercularis_ part of Broca's area | 0.6833 |
|  | 45 - pars triangularis Broca's area | 0.1566 |
|  |  |  |
| CH24 (S12-D8) | 6 - Pre-Motor and Supplementary Motor Cortex | 0.6228 |
|  | 43 - Subcentral area | 0.2847 |
|  | 44 - pars opercularis_ part of Broca's area | 0.0676 |
